# Study of the Effect of Baicalin from *Scutellaria baicalensis* on the Gastrointestinal Tract Normoflora and *Helicobacter pylori*

**DOI:** 10.3390/ijms241511906

**Published:** 2023-07-25

**Authors:** Anastasia Dmitrieva, Oksana Kozlova, Victor Atuchin, Irina Milentieva, Anna Vesnina, Svetlana Ivanova, Lyudmila Asyakina, Alexander Prosekov

**Affiliations:** 1 Laboratory of Natural Nutraceuticals Biotesting, Kemerovo State University, 650043 Kemerovo, Russia; a_piskaeva@mail.ru (A.D.); irazumnikova@mail.ru (I.M.); koledockop1@mail.ru (A.V.); pavvm2000@mail.ru (S.I.); alk_kem@mail.ru (L.A.); 2 Department of Bionanotechnology, Kemerovo State University, 650043 Kemerovo, Russia; ms.okvk@mail.ru; 3 Laboratory of Optical Materials and Structures, Institute of Semiconductor Physics, 630090 Novosibirsk, Russia; 4 Research and Development Department, Kemerovo State University, 650000 Kemerovo, Russia; 5 Department of Industrial Machinery Design, Novosibirsk State Technical University, 630073 Novosibirsk, Russia; 6 R&D Center “Advanced Electronic Technologies”, Tomsk State University, 634034 Tomsk, Russia; 7 Department of General Mathematics and Informatics, Kemerovo State University, 650043 Kemerovo, Russia; 8 Laboratory of Biocatalysis, Kemerovo State University, 650043 Kemerovo, Russia; aprosekov@rambler.ru

**Keywords:** *Scutellaria baicalensis*, baicalin, *Helicobacter pylori*, antimicrobial activity, lactic acid bacteria, symbiosis

## Abstract

The antimicrobial properties of baicalin against *H. pylori* and several probiotic cultures were evaluated. Baicalin was isolated from a dry plant extract obtained by extraction with water at 70 °C. For isolation, extraction was carried out with n-butanol and purification on a chromatographic column. The antimicrobial potential was assessed by evaluating changes in the optical density of the bacterial suspension during cultivation; additionally, the disk diffusion method was used. During the study, the baicalin concentrations (0.25, 0.5, and 1 mg/mL) and the pH of the medium in the range of 1.5–8.0 were tested. The test objects were: suspensions of *H. pylori*, *Lactobacillus casei*, *L. brevis*, *Bifidobacterium longum*, and *B. teenis*. It was found that the greater the concentration of the substance in the solution, the greater the delay in the growth of the strain zone. Thus, the highest antimicrobial activity against *H. pylori* was observed at pH 1.5–2.0 and a baicalin concentration of 1.00 mg/mL. In relation to probiotic strains, a stimulating effect of baicalin (1.00 mg/mL) on the growth of *L. casei* biomass at pH 1.5–2.0 was observed. The results open up the prospects for the use of baicalin and probiotics for the treatment of diseases caused by *H. pylori*.

## 1. Introduction

The normal functioning of the microbiota of the gastrointestinal tract (GIT) depends on the healthy lifespan of the host organism since the microbiota of the GIT protects against the colonization of pathogenic strains, regulates the immune and central nervous systems of the body, etc. [1]. *Helicobacter pylori* is a Gram-negative, pathogenic bacterium that colonizes the gastric mucosa and causes infectious gastrointestinal diseases (chronic gastritis, stomach inflammation, peptic ulcer, stomach cancer, etc.) [2,3]. This bacterium adapts well in an acidic environment [4,5] and has a high virulence due to the production of vacuolating cytotoxin A (VacA) and cytotoxin-associated protein (CagA), etc. [6]. For the treatment and prevention of these diseases, it is necessary to completely destroy (eradicate) *H. pylori* from the gastric mucosa. To date, the generally accepted method of eradication has been the use of triple therapy: taking one or two antibiotics, a proton pump inhibitor, or a bismuth-containing compound [2]. The effectiveness of this method is about 85–90%, but it is gradually decreasing due to the increasing resistance of *H. pylori* to the action of antibiotics and the low pH of gastric juice, and therefore, a high bacterial load [7], as well as due to the occurrence of side effects in patients due to non-compliance with the patient’s treatment regimen [8]. In this regard, the development of new, more effective and safe treatment regimens or the modification of existing ones is relevant. 

A number of scientific papers have presented evidence that the qualitative and quantitative composition of the microbiota of the gastrointestinal tract affects *H. pylori*, preventing the development of diseases induced by this microorganism [9]. Therefore, the intake of probiotic and prebiotic supplements and functional products based on them is one of the effective means of preventing the development of *H. pylori* infection through stimulation and normalization of the gastrointestinal microbiota [1,10,11]. Modern generally accepted methods of the eradication of *H. pylori* have many disadvantages (the occurrence of side effects from the use of antibiotics in consumers, acquired *H. pylori* resistance, etc.). In this regard, it is important to develop more effective, less toxic drugs that will eliminate the shortcomings of traditional treatment regimens. For example, an additional intake of a synbiotic supplement, and functional products based on it, containing probiotics (for example, containing *L. casie*) and baicalin. Studies have confirmed the inhibitory effect of baicalin on *H. pylori*, and stimulation of the growth of the biomass of the probiotic strain *L. casie* under in vitro conditions. These works have made it possible to expand the range of probiotic and synbiotic supplements and functional products based on them, which contribute to the eradication of *H. pylori*. The emphasis on the development of functional food products, for example, dairy and sour milk products (cheese, kefir, etc.), is due to the fact that these products are widely in demand among consumers; they are a good basis for adding probiotics, prebiotics, and synbiotics [12,13].

One of the most promising methods is the additional intake of probiotics, prebiotics, and other biologically active substances (BAS), and functional products based on them, which stimulate the microbiota of the GIT of the host organism and suppress *H. pylori*.

To reduce the side effects from taking antibiotics (eliminate nausea, vomiting, diarrhea, taste disturbance, etc.) [4] and enhance eradication therapy, the additional intake of probiotics is relevant [14,15]. Probiotics are live, non-pathogenic microorganisms that can normalize the work of the microbiota of the gastrointestinal tract and the entire host organism as a whole [16]. It is known that the mechanism of action of a number of probiotic strains on H. pylori is due to the production of peptides and non-peptide substances that inhibit the growth and adhesion of the pathogenic strain [5,17,18]. Probiotics are known to inhibit *H. pylori* attachment to human gastric epithelial cell lines, suppress the inflammatory processes associated with this infection, and inhibit the growth of antibiotic-resistant *H. pylori* [19]. The probiotic microorganisms release antibacterial compounds and short-chain fatty acids, which inhibit the growth of *H. pylori* bacteria. The main short-chain fatty acids include lactic, acetic, and propionic acids, which contribute to a decrease in the pH of the medium [5]. Certain lactobacillus species release protein toxins that are harmful to *H. pylori* strains [20]. *Lactobacillus reuteri* can synthesize non-peptide antipathogenic substances that suppress the expression of the *vacA* and *flaA* genes responsible for *H. pylori* virulence [21]. Microorganisms *L. acidophilus*, *L. bulgaricus*, and *L. rhamnosus* reduce inflammation by reducing the expression of IL-8 in *H. pylori*-infected cells [22]. Studies have shown the ability of *L. rhamnosus* and *L. acidophilus* to inhibit *H. pylori*. As a result, it was found that the analyzed strains exhibited antimicrobial activity, inhibited adhesion to *H. pylori* with MDR, and also stimulated the growth of beneficial microflora (*Bifidobacterium* spp. and *Akkermansia muciniphilia*) in mice. In a study by K. Zhao [23], who studied the activity of *L. plantarum* against *H. pylori*, the strain inhibited the growth, urease activity, and adhesion ability of *H. pylori*. Therefore, the consumption of probiotics helps to reduce the bacterial load of *H. pylori*. In a study by Y. Aiba [24], it was shown that the *L. johnsonii* strain (both in the active and heat-killed state) inhibits *H. pylori*, deforming it. The effectiveness of probiotics can be increased by including prebiotics, biologically active substances that stimulate the work of microorganisms beneficial to the body, in the consumer’s diet. It is known that plants are a promising source of biologically active substances that affect the microflora of the gastrointestinal tract [25].

**Figure 1:**
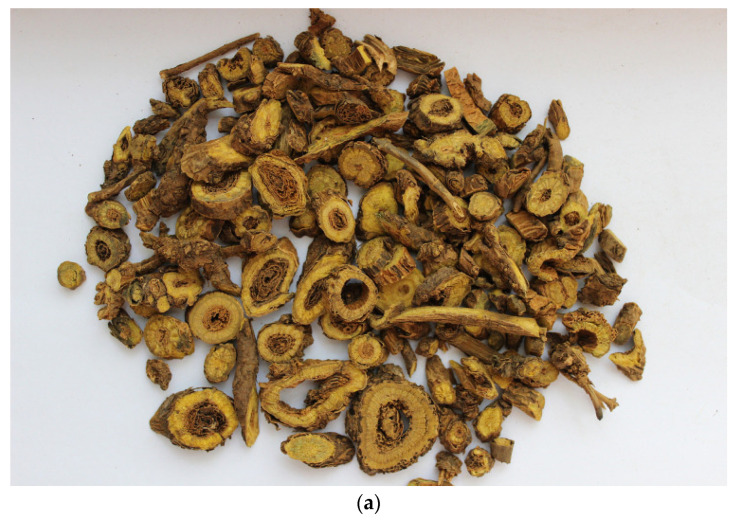

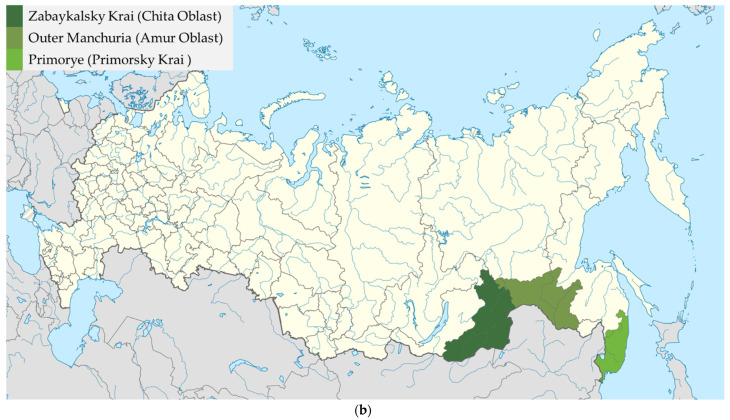
*Scutellaria baicalensis*. (**a**) Plant roots—medicinal part (figure from work L. Xin [30]), (**b**) public cadastral map of Russia, which reflects the habitat of the plant.

The use of medicinal plants in the form of decoctions, infusions, etc., with low toxicity and minor side effects, is an alternative method of eliminating *H. pylori* [4,26,27]. Today, the roots of *Scutellaria baicalensis* [28] are used a Chinese traditional medicinal plant with a high therapeutic potential; they are used to treat gastritis, diarrhea, hepatitis, atherosclerosis, diabetes mellitus, hypertension, eye diseases, vomiting, hemorrhages, insomnia, colds, respiratory infections, etc. [29,30,31,32,33]. In Russia, the plant is distributed in the territories of Zabaykalsky Krai, the Outer Manchuria and southwestern Primorye [34], Figure 1. Its potential is achievable due to the content of secondary metabolites in the plant flavonoids, which are able to protect the body from oxidative stress, showing antimicrobial activity [35]. The main flavonoid of *Scutellaria baicalensis* is baicalin (its concentration in the roots of the plant reaches from 8.1% to 15.6% [30,31]).

Baicalin (7-glucuronic acid, 5,6-dihydroxy-flavone) is a flavonoid compound [36] capable of inhibiting enzymes and regulating the immune response [37,38], exhibiting antitumor, antimicrobial, anti-inflammatory, neuroprotective, antidiabetic, antimutagenic, anticonvulsant, and antioxidant properties [39,40,41]. There are studies in which it has been proven that this flavonoid exhibits antibacterial activity against *H. pylori* [42]: by suppressing the expression of *hefA* genes (a gene affecting the multidrug resistance of *H. pylori*) and *vacA* (a gene that affects the production of exotoxin and promotes bacterial adhesion on stomach cells, leading to vacuolization of the cytoplasm of the target cell, its apoptosis, and death), and by inhibiting the action of urease and increasing the sensitivity of *H. pylori* strains to the action of amoxicillin and tetracycline [43,44,45]. Therefore, it is relevant to use the flavonoid baiсalin as an antimicrobial drug against *H. pylori* [44,46], for example, by adding the use of a synbiotic supplement containing a probiotic and baicalin to the standard eradication therapy. In a study by M. E. Chen [44], baicalin, baicalein, and *L. rhamnosus* were orally administered to mice infected with *H. pylori*. As a result, a synergistic effect was observed, promoting the inhibition of *H. pylori*. X. D. Yu [47] evaluated the ability of baicalin and scutellarin to influence *H. pylori*. As a result, it was found that these polyphenols of *Scutellaria baicalensis* Georgi. suppressed the activity of *H. pylori* by inhibiting urease. Therefore, baicalin, both alone and in combination with probiotics, may be a potential BAS capable of suppressing *H. pylori*.

The analysis of the literature data carried out by these authors showed that a relatively limited number of studies have been presented on the assessment of the effect of baicalin on probiotic strains. Basically, the data are aimed at assessing the impact on representatives of *Lactobacillus* spp. (*L. rhamnosus*, *L. acidophilus*, *L. plantarum*, *L. bulgaricus*, and *L. reuteri*). This work aims at studying the effect of baicalin of various concentrations on *H. pylori* and probiotic strains of the genus *Lactobacillus* spp. (*L. casei*, *L. brevis*) and *Bifidobacterium* spp. (*B. longum*, *B. adolescentis*), which are some of the probiotics on the Russian market [48].

## 2. Results

**Figure 2:**
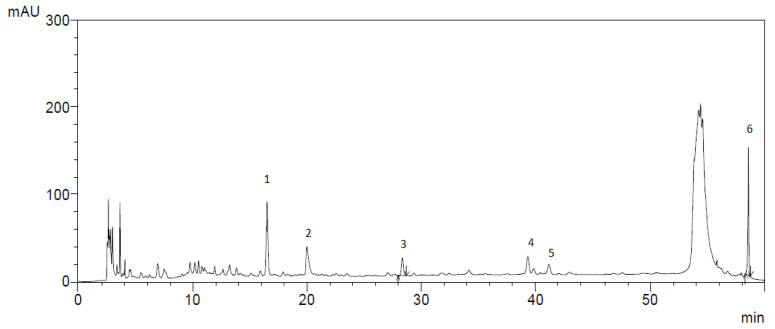
Chromatogram showing the content of BAS in the extract of *Scutellaria baicalensis*: 1—3,4- Dihydroxycinnamic acid; 2—luteolin; 3—rutin; 4—scutellarin; 5—chrysin; 6—baicalin.

The results of the study of the quantitative and qualitative content of biologically active substances (BAS) in the ethanol extract of *Scutellaria baicalensis* are presented in Figure 2 and in Table 1.

**Figure 3:**
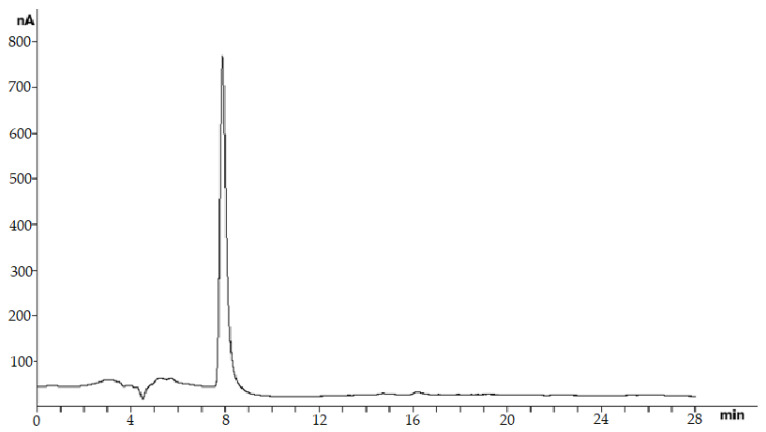
HPLC analysis of baicalin isolated from an ethanol extract of *Scutellaria baicalensis* and purified with Sephadex LH20 using a glassy carbon sensor as an amperometric detector.

**Figure 4:**
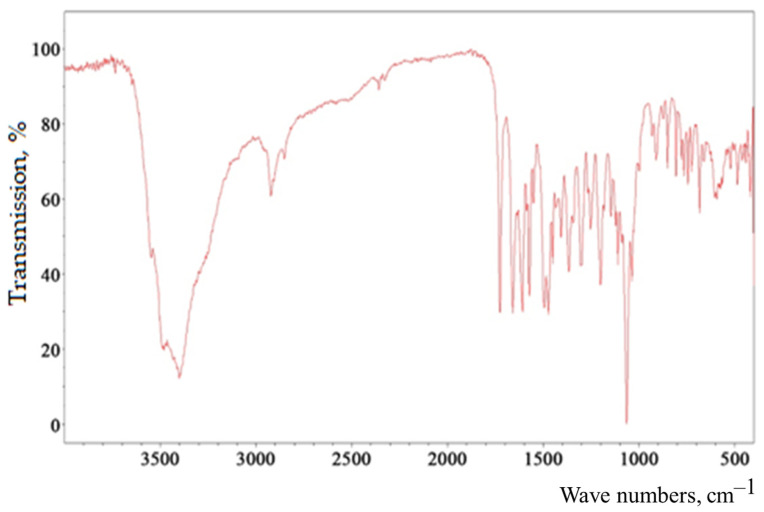
IR spectrum of baicalin isolated from ethanol extract of *Scutellaria baicalensis* and purified with Sephadex LH20.

Chromatographic analysis confirmed the presence of baicalin content in the ethanol extract of *Scutellaria baicalensis* and the expediency of its further isolation and purification. The results of the identification of the obtained purified baicalin (compound purity 97%) isolated from the ethanol extract of *Scutellaria baicalensis* are presented in Figure 3 and Figure 4.

The infrared spectrum (Figure 4) of baicalin (5,6-dihydroxy-4-hydroxy-2-phenyl-4H-1-benzopyran-7-β-D-glucuronide) is characterized by the following features. The band with the absorption maximum at 3398 cm^−1^ is characteristic of the associated OH groups [49,50,51]. Bands at 2922 and 2853 cm^−1^ are found, due to the symmetric and antisymmetric stretching vibrations of the tertiary carbon in the carbohydrate fragment. The presence of C=O in the carboxyl group is characterized by a band at 1725 cm^−1^. The bands at 1657 and 1607 cm^–1^ are due to C=O stretching vibrations, as well as the influence of OH groups in positions three and five of the heterocyclic fragment of the molecule due to the formation of an intramolecular hydrogen bond with C=O. It is the influence of this hydroxyl that causes distortion of the planar arrangement of the pyran fragment and the bond, and thus leads to the appearance of a resonance in the form of a doublet. In turn, OH at the third carbon atom of the unsaturated pyran fragment (ring C) causes a weak band at 1550 cm^−1^. Stretching vibrations of C- bonds in aromatic systems of rings A and B cause bands at 1571, 1496, and 1462 cm^−1^. The OH group at the tertiary carbon is responsible for the 1408 cm^−1^ band. The bands at 1364 and 1304 cm^−1^ appear as a result of O-H in-plane deformation vibrations in the structural components of baicalin. The bands at 1254, 1200, and 1147 cm^−1^ are caused by two interacting antisymmetric vibrations C-O-C and C-C-O in the structures of heterocycles (ring C and carbohydrate fragment). Symmetrical C-O-C and C-C-O stretching vibrations cause bands at 1107 and 1064 cm^−1^ of the carbohydrate fragment. The 910 cm^−1^ band is specific to the pyranose ring. A relatively weak band at 849 cm^−1^ indicates out-of-plane C2-H bending vibrations, thus characterizing the α-anomer with the equatorial arrangement of unsubstituted hydrogen atoms of the B–C ring bond. The trisubstituted ring A is characterized by bands at 788, 764, and 745 cm^−1^ due to deformation out-of-plane and in-plane C-H vibrations. The monosubstituted ring B is characterized by the presence of an out-of-plane bending vibration of the C-C bond at 726 cm^−1^.

**Figure 5:**
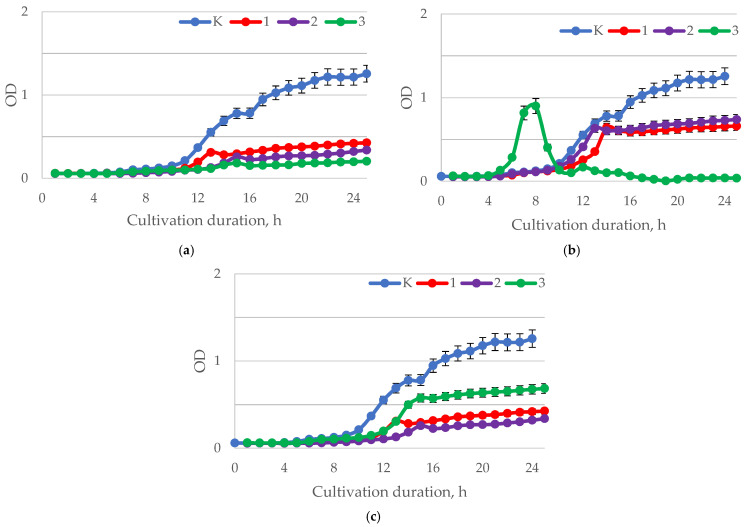
Dynamics of the optical density (λ = 600 nm) of the bacterial suspension of *H. pylori* with different values of active acidity pH (**a**) 1.5–2.0; (**b**) 5.5–6.0; (**c**) 7.5–8.0 with different concentrations of baicalin: K—control (0.00 mg/mL); 1—(0.25 mg/mL); 2—(0.50 mg/mL); 3—(1.00 mg/mL).

The results of the analysis of the antimicrobial activity of baicalin in relation to *H. pylori* are presented in Figure 5.

At pH = 1.5–2.0 (Figure 5a), the introduction of baicalin at the studied concentrations inhibited the growth of *H. pylori* compared to the control. The greatest inhibition of biomass growth was observed when baicalin was added at a concentration of 1.00 mg/mL. In comparison to other samples, samples with baicalin at a concentration of 1.00 mg/mL demonstrated an intensive increase in biomass at pH values of 5.5–6.0 (Figure 5b). The biomass accumulation was suppressed in samples with baicalin concentrations of 0.25 and 0.5 mg/mL. In samples cultivated at pH = 7.5–8.0 (Figure 5c), the addition of baicalin inhibited the growth of *H. pylori* biomass compared to the control. Samples with 0.5 mg/mL baicalin showed the highest inhibitory activity. Table 2 shows the results of the disk diffusion method used to assess *H. pylori* inhibition by baicalin at various concentrations.

**Figure 6:**
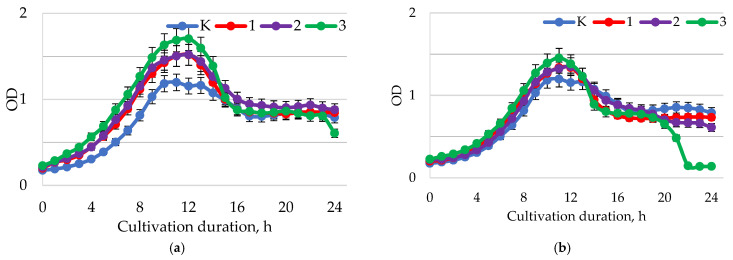

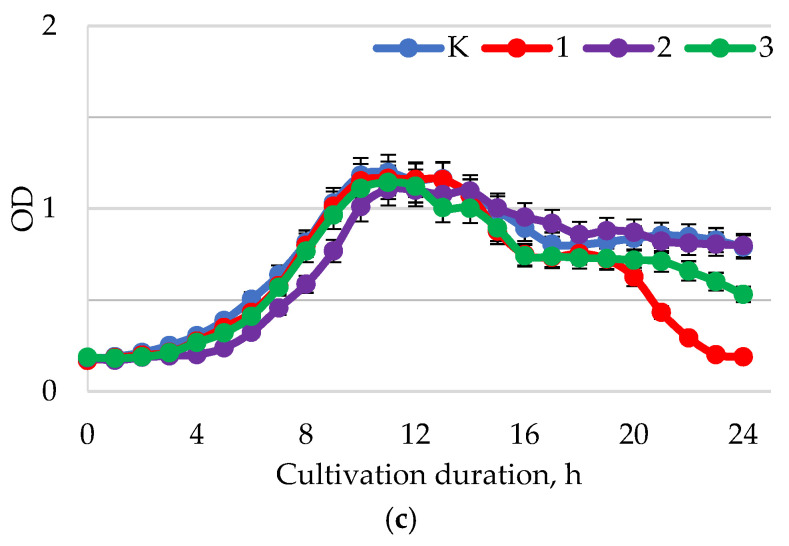
Dynamics of the optical density (λ = 600 nm) of the bacterial suspension of *L. casei* with different values of active acidity pH: (**a**) 1.5–2.0; (**b**) 5.5–6.0; (**c**) 7.5–8.0 with different concentrations of baicalin: K—control (0.00 mg/mL); 1—(0.25 mg/mL); 2—(0.50 mg/mL); 3—(1.00 mg/mL).

**Figure 7:**
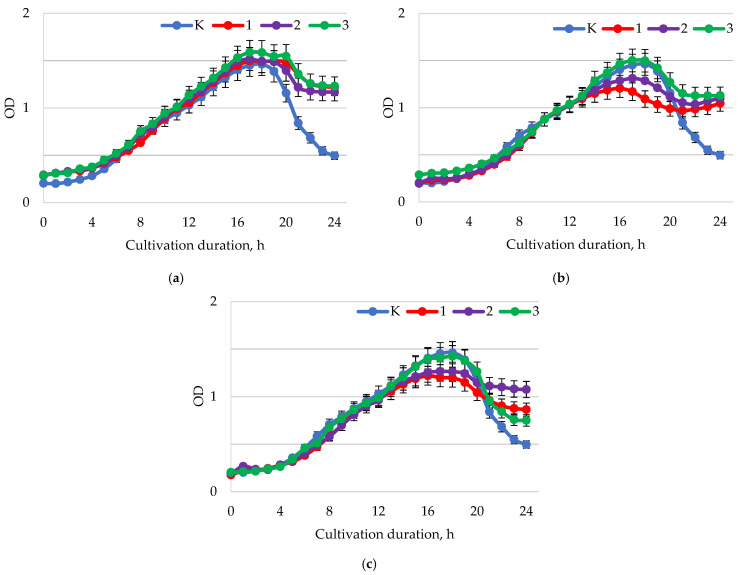
Dynamics of the optical density (λ = 600 nm) of the bacterial suspension of *L. brevis* with different values of active acidity pH: (**a**) 1.5–2.0; (**b**) 5.5–6.0; (**c**) 7.5–8.0 with different concentrations of baicalin: K—control (0.00 mg/mL); 1—(0.25 mg/mL); 2—(0.50 mg/mL); 3—(1.00 mg/mL).

**Figure 8:**
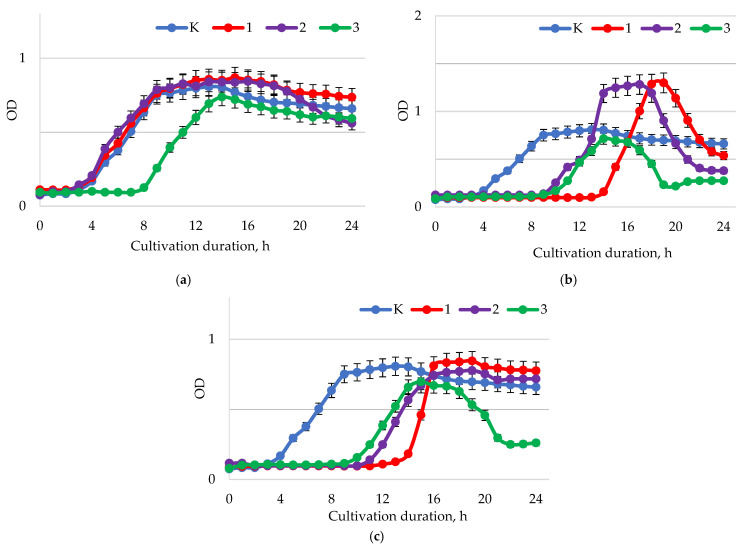
Dynamics of the optical density (λ = 600 nm) of the bacterial suspension of *B. longum* with different values of active acidity pH: (**a**) 1.5–2.0; (**b**) 5.5–6.0; (**c**) 7.5–8.0 with different concentrations of baicalin: K—control (0.00 mg/mL); 1—(0.25 mg/mL); 2—(0.50 mg/mL); 3—(1.00 mg/mL).

**Figure 9:**
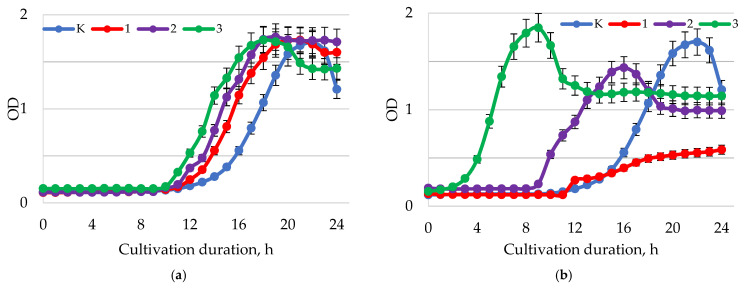

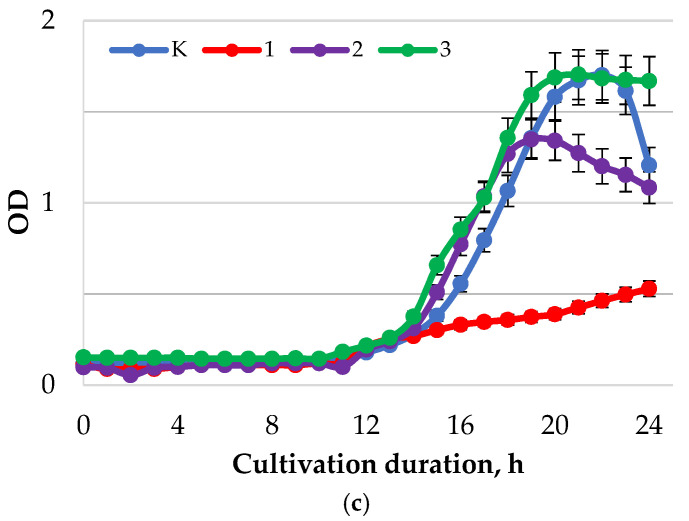
Dynamics of the optical density (λ = 600 nm) of the bacterial suspension of *B. adolescentis* with different values of active acidity pH: (**a**) 1.5–2.0; (**b**) 5.5–6.0; (**c**) 7.5–8.0 with different concentrations of baicalin: K—control (0.00 mg/mL); 1—(0.25 mg/mL); 2—(0.50 mg/mL); 3—(1.00 mg/mL).

In the study, samples of baicalin showed (Table 2) antimicrobial activity against *H. pylori* depending on the concentration. The largest zone of inhibition (18.90 mm) was observed when using samples of baicalin with a concentration of 1.0 mg/mL. Antibiotics (clarithromycin and amoxicillin) were used as controls and they demonstrated higher zones of inhibition of the growth of the pathogenic microorganism. The results of the analysis of the antimicrobial activity of baicalin in relation to representatives of the normal gastrointestinal microflora are presented in Figure 6, Figure 7, Figure 8 and Figure 9.

The highest growth-stimulating activity of baicalin on *L. casei* was observed at pH = 1.5–2.0 (Figure 6a). In this range of pH values, a dose-dependent effect of baicalin on the growth of *L. casei* was recorded. At a concentration of 1.0 mg/mL, the greatest accumulation of biomass occurred since a higher optical density was recorded by the end of the exponential growth phase compared to the control. With an increase in pH to 5.5–6.0 (Figure 5b), the level of their biomass accumulation decreased in comparison with samples of bacterial suspension treated with baicalin at pH = 1.5–2.0. At pH = 7.5–8.0 (Figure 5c), no significant positive effect on the growth and accumulation of bacterial cells was observed.

At a medium pH of 1.5–2.0 (Figure 7a) and 5.5–6.0 (Figure 7a), there were slight positive growth dynamics of the bacterial suspension with *L. brevis* when baicalin was added (Figure 6). The maximum growth of *L. brevis* was observed at a baicalin concentration of 1.0 mg/mL. At pH = 7.5–8.0, *L. brevis* growth was slightly suppressed in this case, as well as in bacterial suspensions with *L. casei*. 

In samples of bacterial suspension containing *B. longum* at pH = 1.5–2.0 (Figure 8a), the effect of baicalin at concentrations of 0.25 and 0.5 mg/mL manifested as a slight increase in biomass accumulation in comparison to the control. These concentrations of baicalin had a growth-stimulating effect. In comparison to the control, there was a smaller increase in the strain biomass over time when baicalin was added to the bacterial suspension at a concentration of 1.0 mg/mL. In the control, the lag phase was observed at 3–4 h of cultivation; for samples with a concentration of 1.0 mg/mL, this was observed only from 9 h. At pH = 5.5–6.0 (Figure 8b), for the control, the lag phase began at about 4 h, for all other samples, later time points occurred. However, despite the delay in the lag phase, the exponential increase in biomass was more pronounced in samples with baicalin concentrations of 0.25 mg/mL and 0.5 mg/mL than in controls. Samples of bacterial suspension with a concentration of 1.0 mg/mL of baicalin showed a delay in the lag phase and a smaller increase in biomass. For pH = 7.5–8.0 (Figure 8c), the best results in terms of biomass growth were shown by samples with a baicalin concentration of 0.25 mg/mL, but with a pronounced delay in the onset of the lag phase (from about 12 h) compared to the control (3.5–4.0 h). All other samples showed a smaller increase in biomass and a delay in the onset of the lag phase.

The growth curves of *B. adolescentis* demonstrate an increase in biomass growth and a decrease in the duration of the lag phase at pH = 1.5–2.0 (Figure 9a). The time of adaptation of microorganisms to the medium decreased depending on the concentration of baicalin. Thus, at doses of 0.25, 0.5, and 1.0 mg/mL, the activation time was about 10–11 h (control—12 h). However, in samples with a baicalin concentration of 1.0 mg/mL, the phase of a decrease in the intensity of bacterial reproduction began earlier (from about 20.5 h). With an increase in the pH of the medium to 5.5–6.0 (Figure 8b), the beginning of the lag phase of bacterial growth changed depending on the concentration of baicalin. At concentrations of 0.5 and 1.00 mg/mL, the adaptation phase was 9.0 and 3.0 h, respectively. At pH 7.5–8.0 (Figure 8c), there were no significant differences in the adaptation time of *B. adolescentis* in the presence of baicalin of various concentrations. 

**Figure 10:**
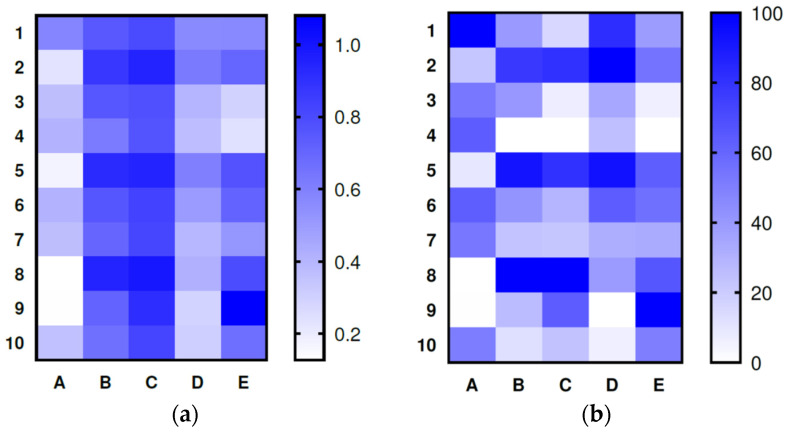
Heat maps of (**a**) absolute and (**b**) normalized mean optical density values of bacterial suspension with test cultures: A—*H. pylori*; B—*L. casei*; C—*L. brevis*; D—*B. longum*; E—*B. adolescentis*.

The results of the statistical processing of data on the effect of baicalin on test cultures (*H. pylori*, *L. casei, L. brevis, B. longum, B. adolescentis*) are presented in Table 3, Figure 10, and Table A1. 

At the initial stage of observation (up to 10 h), no significant differences were found between the samples (*p* > 0.05). With an increase in the duration of observation up to 24 h, the samples significantly differed from the control (*p* < 0.05). At a fixed pH value, no significant differences were found between the samples corresponding to the concentration of baicalin (0.25, 0.50, and 1.00 mg/mL) over the entire observation period, with the exception of sample Y8 (1.00 mg/mL, pH = 5.5–6.0). When the value of active acidity changes, the significance of the effect on the target function of the concentration of baicalin increases. With an increase in the pH value to 7.5–8.0, the influence of the concentration of baicalin decreases; apparently, in this case, the activity of baicalin decreases due to the destructive effect of the pH of the medium. The greatest inhibitory effect on bacterial culture was observed at a pH value of 1.5–2.0 and a baicalin concentration of 1.00 mg/mL.

In general, suppression of *H. pylori* vital activity was observed, and the best indicators were at a pH of 1.5–2.0 and a concentration of baicalin of 1.00 mg/mL. However, the cultivation process requires additional study at a pH of 5.5–6.0 and a concentration of baicalin of 1.00 mg/mL (either there were errors during the conduct, or there were factors we did not take into account but which were significant within the organization of the process). There were no statistically significant differences between the samples of *L. casei* bacterial suspension during the entire observation stage (up to 24 h) (*p* > 0.05). For some modes of cultivation of microorganisms (1.00 mg/mL, pH 5.5–6.0 and 7.5–8.0) in 20 h, a large dynamic of population decrease was observed. Neither the concentration of baicalin nor the pH of the medium had a significant effect on the test culture. However, with some concentration modes at a limited interval (10–16 h), there was a significant difference between the samples and the control (active acidity from 1.5 to 6.0). The phase of the greatest growth of the test culture was observed at the lowest active acidity and the highest concentration of baicalin.

At the incomplete stage of observation (up to 18 h), no statistically significant differences were found between the samples (*p* > 0.05). Neither the concentration of baicalin nor the pH of the medium had a significant effect on the test culture. For most modes of the cultivation of microorganisms in 18 h, significant differences were observed for the dying phase in comparison with the control sample, there were smaller dynamics of population decrease than in the control. The phase of the highest concentration of microorganisms in the medium with the addition of baicalin exceeded from 1 to 3 h compared with that without it. Significant stimulation of *L. brevis* was not observed; the concentration and time of biomass accumulation were approximately the same as without the addition of baicalin, but the concentration stabilization phase lasted longer than the control, 1 up to 3 h (baicalin concentration 1.0 mg/mL, pH 1.5–2.0), which may positively affect the preservation of the quality characteristics of the product during storage and increase the shelf life. 

No significant differences from the control were found for pH 1.5–2.0 samples; the concentration of baicalin was 0.25 and 0.05 mg/mL. In most cases, the number of microorganisms in the bacterial suspension did not exceed the number in the control sample, with the exception of samples obtained at pH 5.5–6.0 and concentrations of baicalin of 0.25 and 0.05 mg/mL; it can be said that in 16 h of observation, the samples obtained at pH 7.5–8.0 did not significantly differ from the control. Intensive accumulation of *B. longum* was not observed; moreover, at a concentration of baicalin of 1.00 mg/mL, suppression of population growth was noted. The exception was the cultivation modes at pH 5.5–6.0 and the concentration of baicalin 0.25–0.05 mg/mL, which need to be studied further, the intensive growth, and the same rapid decline in concentration (something was not taken into account, some kind of impulse effect, and it is necessary to find out what led to different concentration dynamics).

The influence of the studied parameters on the target function is not unambiguous. At a pH value of 1.5–2.0 for all the considered values of the baicalin concentration, the dynamics of the accumulation of microorganisms is similar to the dynamics in the control sample, with a slight acceleration in 12 h of observation and stabilization at a level slightly higher than in the control, in 20 h. With this fixed value of active acidity, no significant differences were found in the samples corresponding to the baicalin concentration values of 0.25, 0.50, and 1.00 mg/mL. At pH 7.5–8.0, at no values of the concentration of baicalin, there was no significant stimulation of biomass accumulation.

Sample Y6 (0.5 mg/mL, pH = 7.5–8.0) requires increased attention; intensive population growth was demonstrated at one concentration of baicalin. Biomass was accumulated at pH 5.5–6.0 almost 2.5 times faster (earlier by 14 h), while at pH 1.5–2.0, it was accumulated earlier by 4–5 h, and at pH 7.5–8.0, it did not significantly differ from the control. It seems that there is a factor that, under these conditions (1.00 mg/mL, pH 5.5–6.0), additionally stimulated the accumulation of biomass, and its effect deserves to be studied. With a fixed value of active acidity at pH 5.5–6.0 at a concentration of baicalin of 0.5 mg/mL, intensive accumulation of biomass was also observed (from 9 to 16 h of observation), but it was not possible to achieve the concentration of the control sample. At pH 5.5–6.0 and pH 7.5–8.0, the baicalin concentration of 0.25 mg/mL led to the inhibition of microorganisms. Basically, there is no stimulating effect of baicalin on *B. adolescentis*, but the cultivation process at pH 5.5–6.0, and a baicalin concentration of 1.00 mg/mL, requires additional study. Intensive and significant population growth has significant prospects for practical application; finding out the mechanism of this simulation to control it is necessary.

## 3. Discussion

This study was aimed at evaluating the effect of different concentrations of baicalin solutions on *H. pylori* and a number of lactic acid bacteria actively used as probiotic supplements. The results obtained showed that baicalin inhibited *H. pylori*; the activity depended on the dose of the substance used. The highest antimicrobial activity against *H. pylori* was observed at pH 1.5–2.0 and a baicalin concentration of 1.00 mg/mL. The obtained data do not contradict the available scientific information. For example, Wu et al. [52] evaluated and compared the antimicrobial activity of baicalin and an alcoholic extract of *Scutellaria baicalensis* against *H. pylori*. Both baicalin and the plant extract were found to have antimicrobial activity against *H. pylori*. MIC50 and MIC90 of baicalin against ten strains of *H. pylori* were 1.04 and 1.30 mg/mL, respectively. In the course of the work carried out, it was found that there was no antimicrobial effect in relation to probiotic strains, and for *L. casei* B 9227, baicalin at a concentration of 1.00 mg/mL, at a pH of 1.5–2.0, had an effect stimulating the growth of biomass. Therefore, the development of synbiotics, functional foods containing baicalin and probiotics (for example, *L. casei*), introduced into the diet with a standard *H. pylori* eradication regimen, is relevant.

This work is relevant, as it expands the list of lactic acid bacteria that suppress *H. pylori*, which in turn expands the range of probiotics, synbiotics, and functional products based on them that are capable of normalizing the beneficial microbiota of the gastrointestinal tract of the host organism, inhibiting urease activity, and, therefore, inhibiting the colonization of *H. pylori*. The advantage of this work is to assess the effect of baicalin solutions of various concentrations not only on *H. pylori* but also on a number of lactic acid bacteria with probiotic activity (by varying the pH of the medium). The lack of work is related to the lack of data on the synbiotic effect of a solution of baicalin and probiotics (*L. casei*, *L. brevis*, *B. longum*, *B. adolescentis*) against *H. pylori*. The disadvantage is planned to be eliminated in the course of further research.

**Figure 11:**
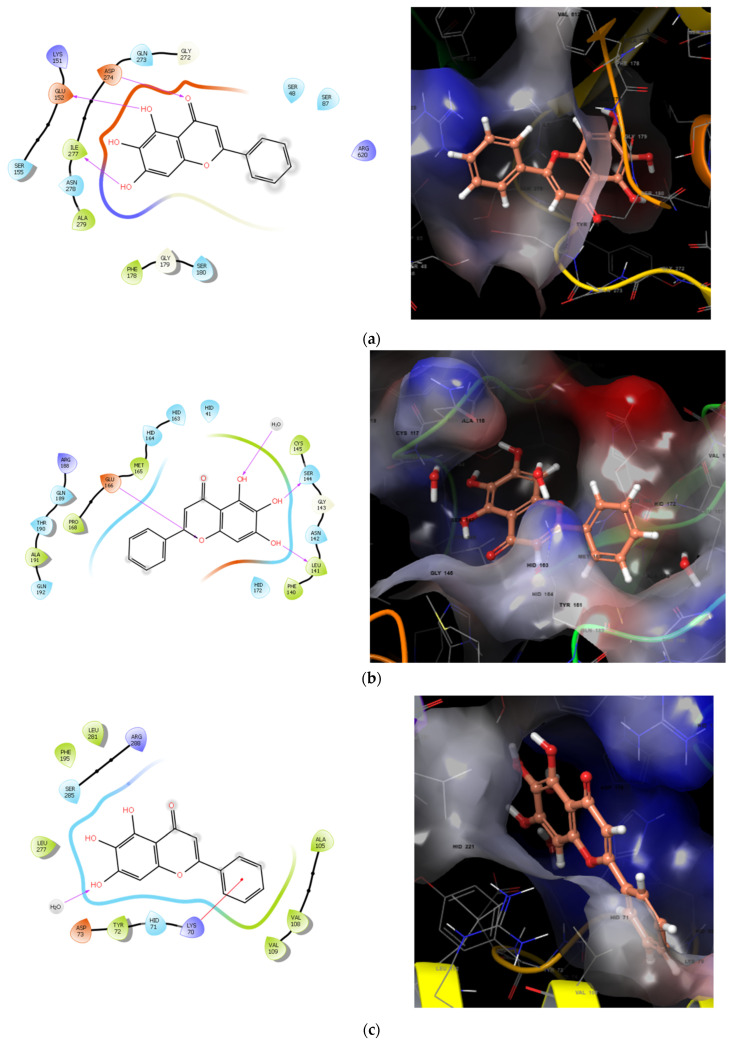

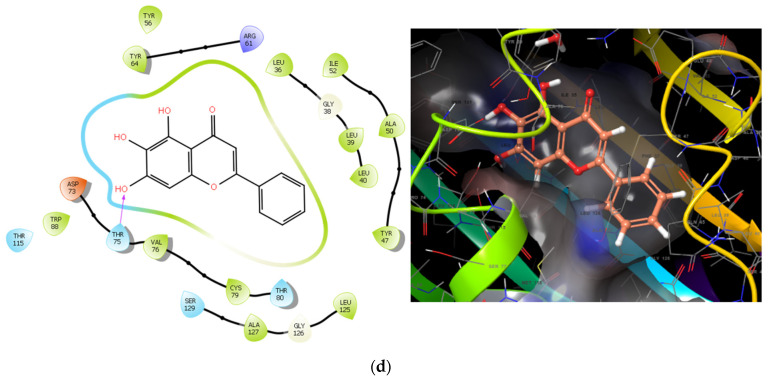
Baicalin and protein binding poses: (**a**)—multidrug exporter MexB 2V50; (**b**)—Sars-CoV protease8CZX; (**c**)—PQSE enzyme 7KGX; (**d**)—transcriptional activator protein PDB code 4NG2.

Molecular docking was performed to predict the molecular interaction of baicalin with test cultures. The Reaxys database was searched for the selection of target proteins. Cases were chosen for baicalin to study activity against proteins important for bacterial growth. We identified critical systems that, when inhibited by baicalin, will prevent bacterial growth and exert an antimicrobial effect. Docking was performed against regulatory protein RhlR (*Escherichia coli*, Wild), transcriptional activator protein LasR (*Escherichia coli*, Wild), an extremely important target of coronavirus 3CLprotease (*Coronavirinae*, Wild) (Figure 11). 

Baicalin preferentially binds to transcription proteins, blocking the synthesis of the RNA of important proteins for the bacterium. On the other hand, there is a rather high affinity of baicalin for the 2V50 multidrug transporter, so baicalin can serve as an adjuvant in the treatment of infections caused by resistant bacteria, including *H. pylori*.

## 4. Materials and Methods

### 4.1. Scutellaria Baicalensis Extraction

The extract was obtained by treating the crushed roots of the *Scutellaria baicalensis* plant with 30% ethanol for 6 h at a temperature of 70 °C in a water bath (WB-6, Daihan Witeg, Republic of Korea) using a reflux condenser [53]. 

### 4.2. Isolation of Polyphenols from the Scutellaria Baicalensis Extracts

Column chromatography was used to determine the quantitative content of polyphenols in the plant extract. A chromatographic column (LC-20 Prominence, Shimadzu, Kyoto, Japan) was filled with a modified sorbent with octadodecyl and terminal aminophenol groups. Polyphenols were eluted with a mixture of water–acetonitrile, 0.1% tributylamine 85:15 was used as an eluent. The elution was carried out in isocratic mode, the flow rate was 1 mL/min, the elution time was 55 min. Detection was performed using a fluorescent detector, the excitation wavelength was 350 nm, the detection wavelength was 415 nm, the collection of individual substances was carried out automatically using a fraction collector [54].

### 4.3. Isolation of Baicalin from the Scutellaria Baicalensis Extracts

The method of extraction of baicalin from the extract of *Scutellaria baicalensis* was taken from the work of Bojko [55]. The isolation of baicalin consisted of: evaporation of ethanol extract under vacuum using a rotary evaporator RV8 (IKA-Werke GmbH & Co., Staufen, Germany) at a temperature not exceeding 50 °C; in addition to the evaporated residue of water heated to 70 °C; the treatment of the aqueous extract with n-butanol, from which baicalin was extracted. Baicalin was purified using gel-penetrating chromatography, using LC-10 chromatograph (Shimadzu, Kyoto, Japan) and Sefadex LH20 (GE Healthcare, Chicago, IL, USA). Additionally, the method of IR spectroscopy using the SF-2000 device (OKB SPECTR, Saint Petersburg, Russia) was used to determine the functional groups in the baicalin molecule. The identification of purified baicalin was carried out using HPLC, the method is presented above. A glassy carbon sensor (UV/VIS detector with a photodiode array with specified wavelengths of 255, 280, 370 nm) was used as a detector. AG Analytekspert, Moscow, Russia, supplied all standards and reagents with purity not less than chemically pure.

### 4.4. Determination of the Antimicrobial Properties of Baicalin

To assess the antimicrobial effect of baicalin on microbial strains, a method was used to assess the increase in biomass by measuring the optical density of suspension cultures. The method was taken from the work of R. Tabasco [56]. In this experiment, during periodic cultivation of strains (at 37 °C), the concentration of baicalin (0.25 mg/mL, 0.50 mg/mL, 1.00 mg/mL) and the pH of the medium were varied (1.5–2.0; 5.5–6.0; 7.5–8.0). The optical density of bacterial suspensions was recorded in time (up to 24 h, with 1 h interval) using a UV 1800 spectrophotometer (Shimadzu, Kyoto, Japan), at a wavelength of λ = 600 nm.

The antibacterial activity of baicalin in vitro was assessed using the disc diffusion method described by H. S. Park [57]. A total of 100 μL of the *H. pylori* suspension optical density (OD) 600 nm = 6.0 for 6 × 10^8^ colony forming units (CFU/mL) was applied to Brucella agar plates supplemented with 10% fetal bovine serum (FBS). A sterile paper disc 6 mm in diameter (TU 2642-001-68085491-2011, Russia) was placed on the surface of the agar, onto which 10 μL of baicalin (0.25; 0.5; 1.0 mg/mL [57,58]) and a positive control, control antibiotics (amoxicillin, clarithromycin), were applied. The plates were incubated at 37 °C for 48 h and the diameter of each zone of inhibition was determined.

The *H. pylori* strain was obtained from the collection the bacteriological laboratory of the Municipal Health Care Institution in the Guryevsky district (Russia). The culture was grown on Mueller-Hinton agar with the addition of 10% defibrinated mutton blood and a selective additive—Campylobacter Selective Supplement (HiMedia, Thane West, India) [57]. Cultivation was carried out in the ILM-170 CO_2_ incubator (LAMSYSTEMS, Miass, Russia) at 37 °C in an atmosphere with 10% CO_2_ [59]. In the experiment, they used an overnight culture grown under similar conditions on a heart–brain broth with the addition of 10% defibrinated mutton blood and Campylobacter Selective Supplement (HiMedia, India).

As probiotic cultures, such strains as *Lactobacillus casei* B-9227, *Lactobacillus brevis* B-10903, *Bifidobacterium longum* AC-1257, *Bifidobacterium adolescentis* AC-1909 were used, they were acquired at (State Research Institute of Genetics and Selection of Industrial Microorganisms of the National Research Center, Moscow, Russia). The strains were previously grown on nutrient media: *Lactobacillus*—on MRS agar (HiMedia, India), and *Bifidobacterium*—on Bifidum medium (Federal Budget Institution of Science, State research Center for Applied Microbiology and Biotechnology, Obolensk, Russia). Cultivation was carried out in the ILM-170 CO_2_-incubator (LAMSYSTEMS, Russia) at a temperature of 37 °C in an atmosphere with 10% CO_2_ [60]. For the experiment, overnight cultures grown on MRS broth (HiMedia, India) were used under the above-described cultivation conditions. Nutrient media without the addition of baicalin, which had different pH values, were used as controls. 

### 4.5. Determination of the Active Acidity of Baicalin Solutions

The method for measuring active acidity (pH) is based on measuring the potential difference between two electrodes (measuring electrode and reference electrode) immersed in the test sample. Measurements were taken with a pH meter (Hanna, Woonsocket, RI, USA). To measure pH, the prepared baicalin solution was placed in a 50 mL beaker and the end of the electrode was immersed in the test solution. It was verified that the electrodes did not touch the glass wall or bottom. The pH value was obtained on the scale of the instrument.

### 4.6. Docking Method

The selection of target proteins for docking was performed in the Reaxys database (reaxys.com, access date 25 January 2023). Protein assemblies were obtained from the PDB database (https://www.wwpdb.org, accessed on 25 January 2023): multidrug exporter MexB 2V50 (PDB DOI: 10.2210/pdb2V50/pdb); Sars-CoV protease 8CZX (PDB DOI: 10.2210/pdb8CZX/pdb); PQSE enzyme 7KGX (PDB DOI: 10.2210/pdb7KGX/pdb); transcriptional activator protein PDB code 4NG2 (PDB DOI: 10.2210/pdb4NG2/pdb). A grid box with dimensions 20 × 20 × 20 and grid spacing 0.375 Å was placed in the active center of corresponding protein. Molecular docking was carried out in Schrödinger Software Suite (Schrödinger, LLC; 2017, New York, NY, USA). 

### 4.7. Statistical Analysis

Statistical analysis to evaluate the effect of baicalin at various concentrations of the extractant, exposure time, and pH of the medium on bacterial cultures was performed using analysis of variance (ANOVA) within the process design module of the Statistica 7.0 program (StatSoft Inc., 2005, Palo Alto, California, USA). Fisher’s test (mean) and Levene’s test (dispersion) were used to assess the homogeneity of the sample distribution. The degree of influence was tested using a post hoc test (LSD test).

## 5. Conclusions

This study evaluated the effect of baicalin isolated from an alcoholic extract of the Baikal skullcap root culture in vitro at various concentrations and pH on the growth of *H. pylori* and some representatives of the normal microflora of the human gastrointestinal tract (*L. casei*, *L. brevis*, *B. longum*, *B. adolescent*). The study confirmed the presence of antimicrobial activity against H. pylori and a stimulating effect on the accumulation of biomass of the normal microflora of the gastrointestinal tract of baicalin, isolated from the extract of the *Scutellaria baicalensis* root. The data obtained indicate that baicalin can be used to treat gastrointestinal diseases caused by *H. pylori* pathogens; however, additional studies are needed to determine the MIC of baicalin in relation to the strains under consideration, as well as to correct the cultivation conditions (confusing peaks, plateaus, etc., on the curves of the dynamics of optical densities, apparently due to the uneven distribution of the accumulated cell biomass, which may be compensated by adding mixing to the technological scheme). In the future, research could be aimed at studying the mechanism of action of baicalin and probiotics in relation to *H. pylori*. For example, the study of the presence/absence of influence on the expression of *hefA*, *vacA*, etc. Research aimed at evaluating the stability of baicalin, baicalin-based supplements, and probiotics under extreme PH conditions are also topical.

## Figures and Tables

**Table 1:** BAS content in the extract of *Scutellaria baicalensis*.

Spike Number	BAS	Molecular Formula	Structural Formula	Retention Time, min	Content, mg/g
1	3,4-Dihydroxycinnamic acid	C_9_H_8_O_4_	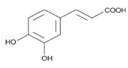	16.28	3.990 ± 0.003
2	Luteolin	C_15_H_10_O_6_	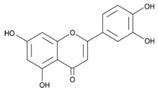	20.03	1.710 ± 0.001
3	Rutin	C_27_H_30_O_16_	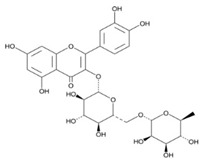	28.35	1.140 ± 0.002
4	Scutellarin	C_15_H_10_O_6_	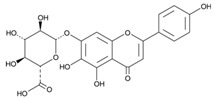	39.18	1.430 ± 0.003
5	Chrysin	C_18_H_12_	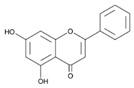	41.10	0.860 ± 0.001
6	Baicalin	C_21_H_18_O_11_	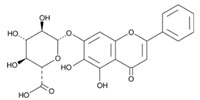	58.53	7.130 ± 0.004

Data presented as a mean or mean ± SD (*n* = 3).

**Table 2:** Antimicrobial activity (zones of *H. pylori* growth inhibition, mm) of baicalin using the disk diffusion method.

Substance	Concentration, mg/mL		
	0.25	0.5	1.0
Baicalin	13.40 ± 0.05 *	15.10 ± 0.05 *	18.90 ± 0.05 *
Clarithromycin	19.60 ± 0.05	29.30 ± 0.05	37.10 ± 0.05
Amoxicillin	24.60 ± 0.05	32.20 ± 0.05	43.50 ± 0.05

Values in a columns followed by the symbol “*” do not differ significantly (*p* < 0.05) as assessed by the post hoc test (Duncan’s test). Data presented as a mean or mean ± SD (*n* = 3).

**Table 3:** Descriptive statistics of value samples of optical density of bacterial suspension.

Sample	Baicalin, mg/mL	pH	Test Culture				
			*H. pylori* (A)	*L. casei* (B)	*L. brevis* (C)	*B. longum* (D)	*B. adolescentis* (E)
Y0	Control		0.5803 ± 0.0135	0.7498 ± 0.0303	0.7996 ± 0.0330	0.5621 ± 0.0236	0.5655 ± 0.0213
Y1	0.25	1.5–2.0	0.2288 ± 0.0104	0.8736 ± 0.0386	0.9467 ± 0.0455	0.6211 ± 0.0281	0.7000 ± 0.032
Y2	0.25	5.5–6.0	0.3668 ± 0.1533	0.7521 ± 0.0337	0.7806 ± 0.0367	0.4030 ± 0.0204	0.2910 ± 0.0140
Y3	0.25	7.5–8.0	0.4136 ± 0.0195	0.6198 ± 0.0301	0.7651 ± 0.0363	0.3714 ± 0.0122	0.2407 ± 0.0188
Y4	0.50	1.5–2.0	0.1678 ± 0.0059	0.9214 ± 0.0391	0.9466 ± 0.0433	0.5982 ± 0.0205	0.7697 ± 0.0302
Y5	0.50	5.5–6.0	0.4120 ± 0.0270	0.7564 ± 0.0352	0.8297 ± 0.0393	0.5010 ± 0.0206	0.7096 ± 0.0344
Y6	0.50	7.5–8.0	0.3680 ± 0.0174	0.6966 ± 0.0342	0.8150 ± 0.0397	0.3935 ± 0.0159	0.5188 ± 0.0251
Y7	1.00	1.5–2.0	0.1266 ± 0.0051	0.9464 ± 0.0434	0.9900 ± 0.0468	0.4183 ± 0.0205	0.7943 ± 0.0372
Y8	1.00	5.5–6.0	0.1270 ± 0.0028	0.7066 ± 0.0360	0.9086 ± 0.0435	0.2887 ± 0.0132	1.0797 ± 0.0484
Y9	1.00	7.5–8.0	0.3570 ± 0.0147	0.6600 ± 0.0303	0.8184 ± 0.0428	0.3091 ± 0.0152	0.6628 ± 0.0327

Data presented as a mean ± SD (*n* = 25).

## Data Availability

The data are available from the authors on request.

## Appendix A

**Table A1:** Results of the dispersion analysis of the dependence of the optical density values of the bacterial suspension on the concentration of baicalin and the pH of the medium.

Samples	*H. pylori*		*L. casei*		*L. brevis*		*B. longum*		*B. adolescentis*	
	I	I*	I	I*	I	I*	I	I*	I	I*
Y1	0.000003	0.084389	0.000005	0.000031	0.000090	0.000001	0.007557	0.000084	0.000000	0.007557
Y2	0.000056	0.133983	0.000006	0.000035	0.000176	0.000006	0.020219	0.000689	0.000000	0.020219
Y3	0.000060	0.128850	0.000002	0.000031	0.000013	0.000003	0.057831	1.000000	0.000000	0.057831
Y4	0.000000	0.060581	0.000022	0.000099	0.000024	0.000001	0.013243	0.005520	0.000000	0.013243
Y5	0.000074	0.143633	0.000004	0.000058	0.000073	0.000002	0.256614	1.000000	0.032460	0.256614
Y6	0.000015	0.130982	0.004144	0.001492	0.000116	0.000001	0.00167	0.000014	0.000000	0.001670
Y7	0.000013	0.180837	0.000000	0.000009	0.000041	0.000001	0.040699	0.000993	0.000045	0.040699
Y8	0.163307	0.557292	0.000005	0.000045	0.000180	0.000001	0.829892	1.000000	0.643357	0.829892
Y9	0.000006	0.080849	0.000028	0.000085	0.000000	0.000003	0.001318	0.039540	0.000000	0.001318

I—LSD test; I*—LSD test (observation period is up to 14 h). Y1—baicalin 0.25 mg/mL, pH = 1.5–2.0; Y2—baicalin 0.25 mg/mL, pH = 5.5–6.0; Y3—baicalin 0.25 mg/mL, pH = 7.5–8.0; Y4—baicalin 0.50 mg/mL, pH = 1.5–2.0; Y5—baicalin 0.50 mg/mL, pH = 5.5–6.0; Y6—baicalin 0.50 mg/mL, pH = 7.5–8.0; Y7—baicalin 1.00 mg/mL, pH = 1.5–2.0; Y8—baicalin 1.00 mg/mL, pH = 5.5–6.0; Y9—baicalin 1.00 mg/mL, pH = 7.5–8.0.
